# The Beilstein Journal of Organic Chemistry and the changing face of scientific publishing

**DOI:** 10.3762/bjoc.11.242

**Published:** 2015-11-18

**Authors:** Martin G Hicks, Peter H Seeberger

**Affiliations:** 1 Beilstein-Institut zur Förderung der Chemischen Wissenschaften, Trakehner Straße 7–9, 60487 Frankfurt am Main, Germany; 2 Max-Planck-Institut für Kolloid- und Grenzflächenforschung, Am Mühlenberg 1, 14476 Potsdam, Germany

Since the launch of the Beilstein Journal of Organic Chemistry in 2005, we have seen many changes that have taken place in scientific publishing. In the beginning, we were one of the first open access journals – now we are one of many. Open access has now become established. However, we are still only one of the few journals offering a completely free service to both authors and readers. The Beilstein Journal of Organic Chemistry has no subscription fees, no page or color charges, no article processing charges (APCs), and is fully peer-reviewed. We were also one of the first to adopt a continuous publishing model for all of our articles, including those belonging to special issues. We created virtual thematic series, each organized by a guest editor and covering a specific topic, where the articles are published as soon as they are ready to ensure short publication times for the authors, and subsequently added to the specific series’ webpage. We have since published over 50 thematic series covering many aspects of organic chemistry. Another innovative addition has been the production of videos filmed directly in the research laboratories of our authors, showing the experiments and linking them to articles published in the journal.

The publishing of scientific papers has several functions: registration, certification, dissemination, inspiring innovation and archiving. Over the last couple of decades, the scientific publishing workflow has become electronic. Nevertheless, all of these developments have not inherently changed the way in which scientific articles have been produced for over three centuries [1]. In the end, the published information ends up on paper – nowadays electronic paper, in the form of a PDF – but with little real innovation or technical progress, and is subsequently abstracted and entered into databases.

In organic chemistry, laboratories are becoming digital in terms of process control, measurement and analytical techniques [2–3]. In terms of publishing, improvements would be beneficial in many areas of structure and data handling and linking. Slowly, the publishing infrastructure and workflows are starting to change. The realization that the way we usually report data is insufficient is growing. Policy statements and communications have been made in the USA and Europe [4–10] and programs have been set up to carry out pilot studies; funding agencies are adapting their policies [11] and universities are implementing them [12]. There are numerous groups working on these issues, for example, the Research Data Alliance and Force11 [13–14]. The changes have generally not yet filtered into scientific journal publishing. The nature of technological advances are such that there is no reason why, for most areas of science (with the exception of a few examples such as the Large Hadron Collider), raw data cannot be captured and stored. The data can be made available at different levels of refinement and processing to meet the differing needs of researchers. Raw data can be made available to experts working directly in a particular field. Further processing will allow it to be stored in structured databases for searching, and with further annotation, it can be linked or added to publications. The Protein Data Bank [15] and the Cambridge Structural Database [16] are two excellent examples of this already well-working concept, and STRENDA DB [17] is a further example nearing release. The next phase in the development of scientific publishing will be the move to make data a central research object in its own right. This will bring about many gains in reproducibility, reuse, efficiency of peer review and publishing. Text and data mining, big data and machine learning, will also become routinely possible, but will only become really useful if the scientific community starts storing and making all verified results – including the negative – publically available. In organic chemistry there is still the general problem of dealing with structure and reaction information in a comprehensive manner. The system should enable chemical structure information to easily adopt two roles simultaneously: well-drawn structure images that are needed for publishing and presentation and also computer-readable formats for validation and searching.

Reading patterns have significantly changed. The number of articles published per year has significantly increased over the last decades, thus routinely “keeping up with the literature” is no longer possible as it was in the past. However, since communication between scientists is now much easier, other methods have replaced this. Now that all journals are produced in electronic format and available online, researchers have largely changed from journal-based browsing and reading to individual article selection, based on search engine results, article alerts or RSS feeds. This has certainly increased usage, since most articles are now accessed and cited, but has changed the way we come across serendipitous findings when browsing, as was previously done by flipping through the pages of a journal. The use of internet search engines and dedicated databases with inherent differences in precision and recall, as well as scientific social media [18], provide good substitutes for traditional browsing.

The problems of plagiarism and self-plagiarism (also known as recycling) [19] are becoming more transparent thanks to open access and the CrossCheck system, to which most scientific publishers (including the Beilstein-Institut), belong. CrossCheck is a database maintained by CrossRef [20] containing all publications of all of its members where new submissions can be checked using the iThenticate system. The use of CrossCheck requires manual control to ensure correct interpretation and for best results is complemented by other search systems and databases. The Beilstein Journal of Organic Chemistry editors have set a level of concordance of around 20% (excluding references, methods and short phrases) as the threshold above which articles are immediately rejected. We have been using this since 2011 for all incoming manuscripts, but to date, we have unfortunately not seen any significant reduction in the number of problematic manuscripts.

One has to question whether it really should be the role of the publisher to police the incoming manuscripts for plagiarism, in particular when often around 20% of submitted manuscripts are rejected based on detected content similarity. Scientific research and the reporting of the results are based on trust. Plagiarism and recycling are seriously diluting the quality of science and are a significant waste of time [19]. In this regard, we believe that the situation has become unacceptable and the scientific community as a whole should become more active. Currently, the detection of these issues takes place behind closed doors; increased transparency could help. Perhaps journals should simply stop checking and allow CrossCheck to carry out a comprehensive post-publishing text-matching analysis, which could be followed by open publication of the results. That this suggestion has not found much support is probably not surprising, but it would be a very interesting exercise and might help reduce the number of problems.

The past ten years were all about technical changes and the broad acceptance of open access – in this regard, the Beilstein Journal of Organic Chemistry fundamentally contributed and helped to advance the field. What the next ten years will bring is hard to imagine. On one hand, quality publications of high impact will continue to flourish. At the same time, many of the open access journals that sprung up over that past decade but contribute little are expected to fall by the wayside. Given that the number of active scientists is still increasing year-to-year, we probably have not yet reached “peak journals”. A more radical change is already underway in how we communicate data and the role that journals will play in the future. Journal articles in their present form play an important role in the rewards and promotions system. Is it the role of journal editors combined with impact factors to decide what is the best science [21]? Wouldn’t it be better for scientists to read about science and make promotion decisions based on sound knowledge? “Living” scientific articles in the form of blogs or similar to Wikipedia would enable reporting of research as it develops and present one complete story rather than fragments.

Technically, a fundamental change is possible. Whether it is desirable for the scientific community, that to an ever-larger extent relies on impact factors, citations, H-indices and the like, is questionable. Here funding agencies are key in terms of their future requirements and their open data policy. Furthermore, the way credit is given and for what needs to change. For example, scientists should receive as much credit for publishing good data sets as they currently do for good articles in a high-impact journal. The Beilstein Journal of Organic Chemistry will aim to help bring about a focus on science and data, and thereby, a back-to-basics approach.
